# Development and validation of a prediction model for microvascular complications of type 2 diabetes based on inflammation-metabolism composite indicators

**DOI:** 10.3389/fendo.2026.1769722

**Published:** 2026-04-14

**Authors:** Yuting Li, Minawaer Hujiaaihemaiti

**Affiliations:** General Practice Department, The First Affiliated Hospital of Xinjiang Medical University, Xinjiang, China

**Keywords:** diabetes, inflammatory indicators, metabolic indicators, microvascular complications, model construction, risk assessment

## Abstract

**Objective:**

This study aimed to evaluate the clinical utility of novel inflammatory and metabolic composite indices in early risk prediction of microvascular complications in patients with type 2 diabetes mellitus (T2DM), and to provide reliable evidence for early precision risk stratification.

**Methods:**

A retrospective analysis was conducted on 964 hospitalized patients with T2DM admitted to the Department of Endocrinology, First Affiliated Hospital of Xinjiang Medical University, from September 2023 to March 2025. Patients were randomly assigned to a training cohort and a validation cohort at a ratio of 7:3 using a random number table. In the training cohort, least absolute shrinkage and selection operator (LASSO) regression was applied for variable selection and to reduce multicollinearity, followed by univariate and multivariate logistic regression analyses to identify independent risk factors for T2DM related microvascular complications. Receiver operating characteristic (ROC) curves, calibration curves, and decision curve analysis (DCA) were employed to comprehensively assess the predictive performance and clinical utility of the model.

**Result:**

Multifactorial logistic regression analysis showed that age, duration of diabetes, duration of hypertension, urine albumin-to-creatinine ratio (UACR) > 30 mg/g, as well as core indicators SIRI and TyG index, were significantly associated with the occurrence of microvascular complications in type 2 diabetes mellitus (T2DM) (P < 0.05). The predictive model constructed based on LASSO-logistic regression demonstrated an AUC of 0.869 (95% CI: 0.842-0.895) in the training set and an AUC of 0.864 (95% CI: 0.824-0.905) in the validation set, indicating stable and excellent discriminatory ability.

**Conclusion:**

This study confirms that SIRI and TyG index can serve as independent risk factors for microvascular complications in T2DM. The nomogram model constructed based on LASSO-logistic regression shows significantly better predictive performance than single indicators, with good model calibration, demonstrating excellent clinical net benefit. This model can accurately assess the risk of microvascular complications, providing reliable decision support for early clinical screening and risk stratification management.

## Highlights

- The analysis results based on LASSO regression and multifactorial logistic regression indicate that age, duration of diabetes, duration of hypertension, UACR, SIRI, and TyG index are independent risk factors for microvascular complications of type 2 diabetes.
- The inflammation-metabolism composite index nomogram model constructed based on LASSO-Logistic regression significantly outperforms single indicators in predicting the risk of microvascular complications, demonstrating good clinical application value across different risk thresholds.

## 1 Introduction

Type 2 Diabetes Mellitus (T2DM) is a global metabolic disease characterized primarily by insulin resistance (IR) and impaired pancreatic β-cell function. Its prevalence continues to rise due to an aging population, the obesity epidemic, and changes in lifestyle. According to the latest data from the International Diabetes Federation (IDF), approximately 589 million adults aged 20 to 79 worldwide are living with diabetes in 2024, with projections suggesting this number will increase to 853 million by 2050 (1). The main dangers of T2DM stem from chronic complications, particularly microvascular complications such as Diabetic Retinopathy (DR), Diabetic Kidney Disease (DKD), and Diabetic Peripheral Neuropathy (DPN), which can lead to blindness, end-stage renal disease, and nerve damage, making them significant causes of disability and mortality among patients (2, 3). Due to the early insidious nature and atypical symptoms of microvascular complications, there is an urgent clinical need for sensitive, cost-effective, and scalable early risk prediction methods.

Currently, the common clinical assessment indicators for complications of Type 2 Diabetes Mellitus (T2DM) primarily focus on blood glucose-related metrics. For instance, glycated hemoglobin (HbA1c) reflects the average blood glucose level of patients over the past three months and is frequently used for diabetes diagnosis and glucose control (4). However, an increasing body of evidence suggests that HbA1c has limitations in predicting microvascular complications: on one hand, HbA1c is influenced by factors such as anemia, hemoglobin variants, and ethnicity, which prevents it from fully reflecting blood glucose fluctuations (5),on the other hand, solely relying on HbA1c levels does not completely exclude the risk of microvascular complications (6). In recent years, with the deepening of research into pathological mechanisms, scholars have discovered that chronic low-grade inflammation plays a crucial role in the occurrence and development of microvascular complications in T2DM, interwoven with metabolic disorders, jointly driving the progression of lesions (7).

In terms of inflammation, prolonged hyperglycemia can activate the pathways of advanced glycation end-products (AGEs) and oxidative stress, inducing inflammatory cells to release various cytokines, damaging vascular endothelium and exacerbating immune imbalance (8). The neutrophil-to-lymphocyte ratio (NLR), monocyte-to-lymphocyte ratio (MLR), systemic inflammation response index (SIRI), and systemic immune-inflammation index (SII) based on routine blood tests can integrate changes in different inflammatory cell subtypes, sensitively reflecting systemic inflammation levels, and possess good accessibility and clinical promotion value (9). In terms of metabolism, lipid metabolism disorders and insulin resistance promote atherosclerosis and abnormalities in blood glucose and lipids (10, 11). Composite indicators such as atherogenic index of plasma (AIP), triglyceride-glucose index (TyG), and TyG-BMI can quantify metabolic abnormalities from multiple dimensions, with existing studies confirming their significant association with microvascular complications (12–14).

Although previous studies have reported the associations of inflammatory or metabolic indicators with diabetic microvascular complications (DMC) separately, there is still insufficient systematic integration of “inflammation-metabolism” composite indicators, and existing predictive models have rarely incorporated such multidimensional information. Therefore, this study aims to retrospectively collect relevant data, utilize LASSO regression to screen for key variables, and construct a nomogram prediction model based on a multidimensional index of ‘inflammation-metabolism-clinical’ factors. It will systematically evaluate the predictive value of inflammatory composite indicators (NLR, MLR, SIRI, SII) and metabolic composite indicators (AIP, TyG, TyG-BMI) in microvascular complications of T2DM. The model will be assessed and validated in both the training and validation sets, with the hope of providing new clinical tools for early risk stratification and precise intervention in microvascular complications of T2DM.

## 2 Materials and methods

This study implemented strict quality control, employed multiple imputation methods to address missing data, and utilized Bootstrap resampling to validate the stability of regression coefficients, thereby ensuring the interpretability of the model’s predictive results and their clinical reliability.

### 2.1 Time and place

This study is a single-center retrospective study, which included patients with Type 2 Diabetes Mellitus (T2DM) who visited the Department of Endocrinology at the First Affiliated Hospital of Xinjiang Medical University from September 2023 to March 2025. All experimental procedures were carried out strictly in accordance with the relevant ethical guidelines and regulations. This research has been approved by the Ethics Committee of the First Affiliated Hospital of Xinjiang Medical University, and was conducted in strict accordance with the ethical standards stipulated by the committee (Ethical Review Number:K202602-12). The patient voluntarily participated in this study and signed the informed consent form.

### 2.2 Inclusion and exclusion criteria

#### 2.2.1 Inclusion criteria

(i) The diagnostic criteria for diabetes mellitus are in accordance with the “Guidelines for the Prevention and Treatment of Type 2 Diabetes in China (2024 Edition)” (15). (ii) Diabetic Retinopathy (DR) conforms to the international clinical grading standards for diabetic retinopathy (16). (iii)Diabetic Kidney Disease (DKD): According to the diagnostic criteria set by the ADA in 2020 (17). (iv) Diabetic Peripheral Neuropathy (DPN) adheres to the expert consensus on the diagnosis and treatment of diabetic neuropathy from the 2021 edition (18). (v) Complete relevant data, including demographic characteristics, medical history, and laboratory indicators (blood routine, biochemistry, blood lipids, blood glucose). This study received ethical approval from the Institutional Review Board of the First Affiliated Hospital of Xinjiang Medical University.

#### 2.2.2 Exclusion criteria

(i) Patients with type 1 diabetes, gestational diabetes, and specific types of diabetes (such as MODY and secondary diabetes); (ii) Those with acute complications (such as diabetic ketoacidosis and hyperglycemic coma) or in a state of acute stress due to infections, trauma, or surgery; (iii) individuals with concomitant malignant tumors, autoimmune diseases (such as rheumatoid arthritis or systemic lupus erythematosus), chronic liver diseases (such as cirrhosis or severe hepatitis), or end-stage renal disease (post-dialysis or kidney transplantation); (iv) individuals who have used immunosuppressants, glucocorticoids, or medications that may affect inflammatory/metabolic markers (such as specific lipid-lowering agents or non-insulin sensitizers) within the past three months.

### 2.3 Sample size calculation

This study conducted a *post hoc* sample size estimation based on the Riley framework (19), including 964 patients (of which 477 experienced events), with an Events Per Variable (EPV) as high as 79.5. This sample size far exceeds the ‘10 EPV’ criterion and the requirements of the Riley framework for shrinkage coefficients (≥0.9) and absolute errors in R² (≤0.05), effectively mitigating overfitting and ensuring the robustness of the model.

### 2.4 Observation indicators

Data was collected from patients through the hospital’s electronic medical record system, including the following information: gender, age, duration of diabetes, history of hypertension, smoking history, alcohol consumption history, and Body Mass Index (BMI). All patients had peripheral venous blood collected the following morning after fasting for ≥8 hours. The measured indicators included: Neutrophil count (NE), Lymphocyte count (LYM), Monocyte count (MONO), Hemoglobin (Hb), Red blood cell distribution width (RDW), Platelet count (PLT), Glycated hemoglobin (HbA1c), and Fasting blood glucose (FBG), Serum creatinine (Scr), serum uric acid (SUA), blood urea nitrogen (BUN), triglyceride (TG), total cholesterol (TC), high-density lipoprotein cholesterol (HDL-C), low-density lipoprotein cholesterol (LDL-C), serum albumin (Alb), alanine aminotransferase (ALT), aspartate amino transferase (AST), Vitamin D(VitD), osteocalcin (OC), and parathyroid hormone (PTH).

### 2.5 Statistical methods

**Figure 1:**
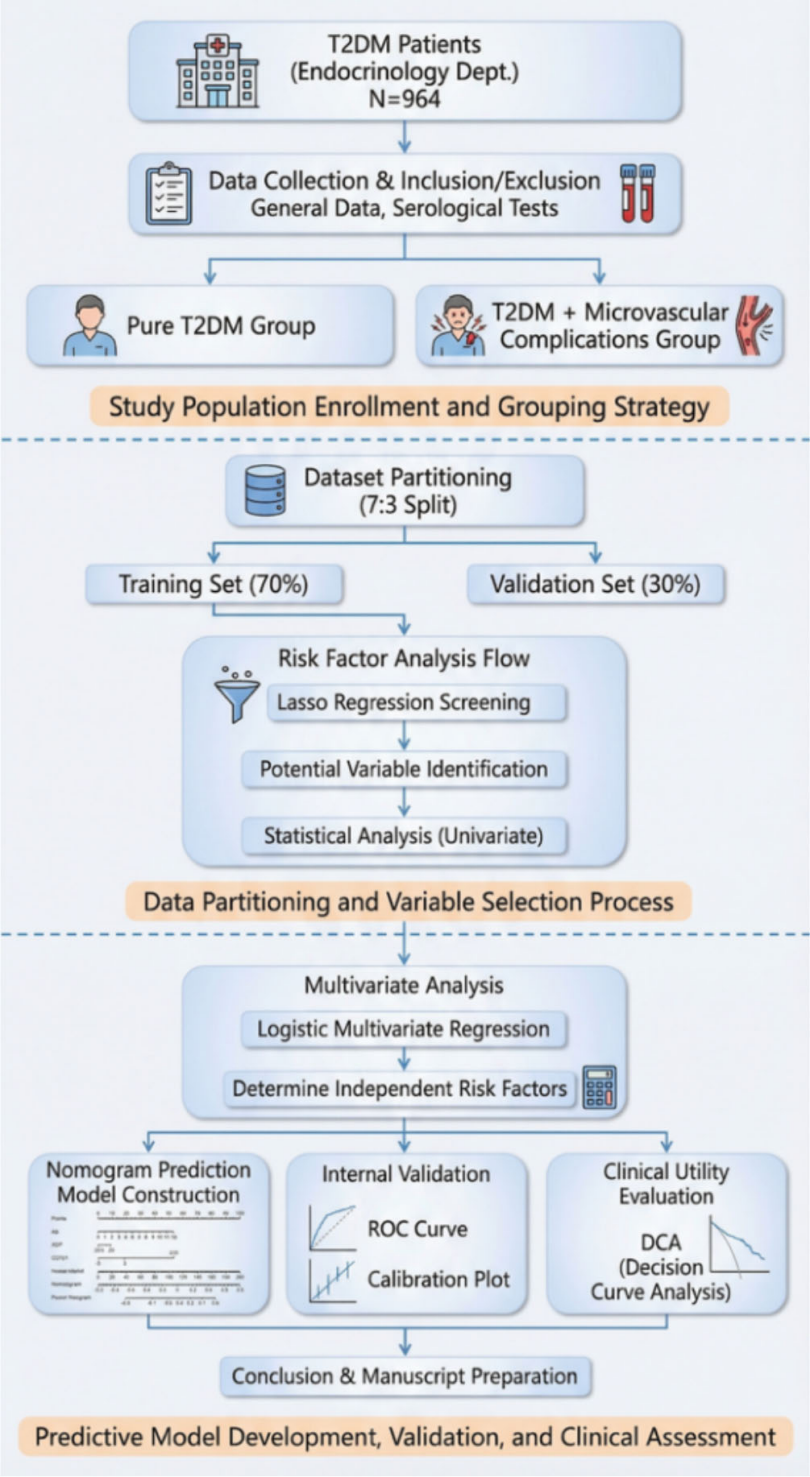
Technical workflow diagram.

In this study, R version 4.3.0 was utilized to randomly divide 964 subjects into training and validation sets at a ratio of 7:3. Baseline data were compared using t-tests, Mann-Whitney U tests, or χ² tests according to the data types. In the training set, LASSO regression and variance inflation factor (VIF) were employed for preliminary screening and exclusion of collinear variables. Subsequently, univariate and multivariate stepwise logistic regression were used to identify independent influencing factors and construct a nomogram prediction model. Finally, the model was comprehensively validated using ROC curves to evaluate discrimination, calibration curves and the Hosmer-Lemeshow test for assessing consistency, as well as decision curve analysis (DCA) for evaluating clinical net benefits across both datasets (two-sided test P<0.05 was considered statistically significant). **Figure 1** presents the technical roadmap of this article.

## 3 Research results

### 3.1 Comparison and analysis of baseline data of the research subjects

This study included a total of 964 patients (**Table 1**) with type 2 diabetes, who were randomly divided into a training set (n=674) and a validation set (n=290) in a 7:3 ratio. The balance test in **Table 1** shows that the two groups in terms of demographic characteristics, disease duration, lifestyle, multiple laboratory indicators (including liver and kidney function, inflammation and metabolism-related indicators, etc.), and the prevalence of microvascular complications (DN, DR, DPN) (all P>0.05). The results suggest that the baseline data distribution between the training set and the validation set is balanced and has good comparability. **Table 2** presents the calculation formulas for the inflammatory and metabolic composite indicators.

**Table 2:** Indicator calculation formulas.

Serial number	Formula
(1)	NLR= NE / LYM
(2)	MLR= MONO / LYM
(3)	SIRI = NE×MONO / LYM
(4)	SII = NE×PLT / LYM
(5)	AIP = log_10_ [TG(mmol/L)/ HDL-C(mmol/L)]
(6)	TyG=ln[(TG(mg/dL)×FBG(mg/dL)/2]
(7)	TyG-BMI= TyG × BMI
(8)	BMI= Weight(kg)/ Height(m)²

**Table 1:** Baseline data of the training set and validation set.

Variable	Overall (n = 964)	Validation set (n = 290)	Training set (n = 674)	Statistical quantity	*P*
Age	53.53 ± 11.93	54.56 ± 11.56	53.09 ± 12.06	t=1.76	0.079
Duration of Hypertension	0.00 (0.00, 6.00)	0.00 (0.00, 5.75)	0.00 (0.00, 6.00)	Z=-0.08	0.934
Gender				χ²=0.93	0.335
Man	561 (58.20)	162 (55.86)	399 (59.20)		
Woman	403 (41.80)	128 (44.14)	275 (40.80)		
Duration of Diabetes				χ²=0.38	0.539
<10 years	621 (64.42)	191 (65.86)	430 (63.80)		
≥10 years	343 (35.58)	99 (34.14)	244 (36.20)		
Hypertension				χ²=0.04	0.835
No	560 (58.09)	167 (57.59)	393 (58.31)		
Yes	404 (41.91)	123 (42.41)	281 (41.69)		
Smoking				χ²=0.71	0.400
No	663 (68.78)	205 (70.69)	458 (67.95)		
Yes	301 (31.22)	85 (29.31)	216 (32.05)		
Alcohol				χ²=3.53	0.060
No	758 (78.63)	239 (82.41)	519 (77.00)		
Yes	206 (21.37)	51 (17.59)	155 (23.00)		
Hb (g/L)	141.00 (131.00, 152.00)	141.00 (131.00, 153.00)	142.00 (130.25, 152.00)	Z=-0.31	0.753
HBW (%)	12.60 (12.20, 13.20)	12.70 (12.20, 13.20)	12.60 (12.20, 13.17)	Z=-0.80	0.425
HbA1C (%)	8.22 (6.90, 9.99)	8.10 (6.88, 9.95)	8.30 (6.91, 10.00)	Z=-0.77	0.441
BUN (mmol/L)	5.90 (4.99, 7.10)	5.94 (5.01, 7.20)	5.89 (4.90, 7.00)	Z=-1.07	0.285
Scr (umol/L)	64.15 (54.00, 76.05)	64.47 (53.18, 75.57)	63.65 (54.10, 76.20)	Z=-0.15	0.884
SUA (umol/L)	303.55 (251.12, 364.85)	301.60 (255.85, 361.57)	303.95 (249.72, 366.12)	Z=-0.04	0.968
Alb (g/L)	40.90 (38.70, 43.10)	41.15 (39.12, 43.10)	40.72 (38.51, 43.00)	Z=-1.36	0.173
AST (U/L)	20.60 (17.10, 27.51)	20.10 (16.92, 26.42)	20.74 (17.20, 27.90)	Z=-1.51	0.131
ALT (U/L)	21.48 (15.90, 32.35)	20.80 (16.26, 32.27)	21.66 (15.82, 32.45)	Z=-0.40	0.691
UACR(>30mg/g)	9.20 (5.42, 19.40)	9.41 (5.41, 19.97)	9.10 (5.42, 19.24)	Z=-0.18	0.859
VitD (nmol/L)	42.19 (31.19, 56.52)	42.53 (32.03, 56.56)	42.12 (30.68, 56.44)	Z=-0.51	0.612
OC (ng/mL)	12.50 (9.61, 15.74)	12.41 (9.52, 15.75)	12.52 (9.66, 15.70)	Z=-0.19	0.849
PTH (pmol/L)	3.52 (2.69, 4.65)	3.60 (2.68, 4.81)	3.50 (2.69, 4.59)	Z=-0.56	0.573
NLR	1.69 (1.29, 2.20)	1.75 (1.31, 2.22)	1.68 (1.27, 2.19)	Z=-1.04	0.296
MLR	0.23 (0.19, 0.29)	0.24 (0.18, 0.29)	0.23 (0.19, 0.30)	Z=-0.14	0.890
SIRI	0.85 (0.58, 1.20)	0.85 (0.58, 1.19)	0.85 (0.59, 1.21)	Z=-0.06	0.955
SII	381.89 (285.27, 528.23)	391.49 (296.29, 519.60)	378.36 (282.59, 528.45)	Z=-0.55	0.581
NE (10^^9^/L)	3.61 (2.88, 4.42)	3.60 (2.91, 4.46)	3.62 (2.83, 4.41)	Z=-0.35	0.730
LYM (10^^9^/L)	2.09 (1.71, 2.61)	2.04 (1.64, 2.64)	2.09 (1.72, 2.59)	Z=-0.95	0.344
MONO (10^^9^/L)	0.49 (0.40, 0.60)	0.49 (0.40, 0.60)	0.50 (0.41, 0.61)	Z=-1.43	0.154
PLT (10^^9^/L)	226.50 (196.00, 268.00)	227.00 (194.00, 263.00)	226.00 (196.00, 271.00)	Z=-0.56	0.576
AIP	0.24 (0.04, 0.45)	0.24 (0.03, 0.44)	0.24 (0.04, 0.46)	Z=-0.28	0.781
TyG	9.19 (8.69, 9.77)	9.18 (8.66, 9.76)	9.20 (8.70, 9.77)	Z=-0.15	0.881
TyG-BMI	239.36 (209.16, 274.59)	240.24 (208.62, 277.53)	238.28 (209.50, 271.80)	Z=-0.55	0.585
BMI(kg/m^2^)	26.00 (23.00, 28.00)	26.00 (23.00, 29.00)	26.00 (23.00, 28.00)	Z=-0.39	0.694
FBG(mmol/L)	7.37 (5.91, 9.38)	7.41 (5.88, 9.59)	7.33 (5.91, 9.33)	Z=-0.08	0.936
TG(mmol/L)	1.62 (1.10, 2.52)	1.60 (1.10, 2.55)	1.63 (1.10, 2.50)	Z=-0.16	0.872
TC(mmol/L)	4.30 (3.52, 5.06)	4.26 (3.52, 4.96)	4.33 (3.51, 5.10)	Z=-0.56	0.579
HDL(mmol/L)	0.97 (0.82, 1.13)	0.96 (0.83, 1.14)	0.97 (0.82, 1.13)	Z=-0.35	0.729
LDL(mmol/L)	2.87 (2.28, 3.44)	2.84 (2.29, 3.41)	2.90 (2.27, 3.47)	Z=-0.60	0.547
DKD				χ²=0.28	0.595
No	833 (86.41)	248 (85.52)	585 (86.80)		
Yes	131 (13.59)	42 (14.48)	89 (13.20)		
DR				χ²=0.00	0.959
No	524 (54.36)	158 (54.48)	366 (54.30)		
Yes	440 (45.64)	132 (45.52)	308 (45.70)		
DPN				χ²=0.02	0.894
No	552 (57.26)	167 (57.59)	385 (57.12)		
Yes	412 (42.74)	123 (42.41)	289 (42.88)		

**Table 3** compares the baseline data between the complication group and the simple T2DM group. There were no statistically significant differences in gender, smoking history, alcohol consumption history, glycosylated hemoglobin, uric acid, AST, total cholesterol (TC), high-density lipoprotein (HDL), and low-density lipoprotein (LDL) (P > 0.05). In terms of demographics and disease duration, the complication group was significantly older than the simple group (p<0.001), and vitamin D levels were significantly higher in the complication group (all P < 0.05). In terms of calcium and phosphorus metabolism indicators, the levels of osteocalcin and parathyroid hormone in the complication group were significantly lower than those in the simple group (all P < 0.001). Inflammatory-related indicators showed a significant increase in the complication group, with NLR, MLR, SIRI, and SII all having P < 0.001. Neutrophil and monocyte counts were significantly elevated, while lymphocyte and platelet counts were significantly reduced (all P < 0.05). Regarding metabolic-related indicators, the TyG index, TyG-BMI, fasting blood glucose, and triglyceride (TG) levels in the complication group were significantly higher than those in the simple group (all P < 0.05 or P < 0.001). In terms of the occurrence of microvascular complications, the non-diabetic nephropathy group showed no incidence of diabetic nephropathy, retinopathy, or neuropathy, while the complication group had prevalence rates of 26.57%, 91.94%, and 86.27%, respectively. The differences between the two groups were statistically significant (P < 0.001 for all), indicating that the abnormal elevation of inflammatory markers and the disorder of metabolic indicators are closely related to the occurrence of microvascular complications in T2DM.

**Table 3:** Comparison of baseline data between the T2DM complication group and the simple diabetes group within the training set.

Variable	Overall (n = 674)	DMC group (n = 335)	simple diabetes (n = 339)	Statistical quantity	*P*
Age	53.09 ± 12.06	56.56 ± 11.18	49.66 ± 11.94	*t* = 7.75	<0.001
Gender				*χ²* = 3.15	0.076
Man	399 (59.20)	187 (55.82)	212 (62.54)		
Woman	275 (40.80)	148 (44.18)	127 (37.46)		
Duration of Diabetes				*χ²* = 88.62	<0.001
<10 years	430 (63.80)	155 (46.27)	275 (81.12)		
≥10 years	244 (36.20)	180 (53.73)	64 (18.88)		
hypertension				*χ²* = 30.48	<0.001
No	393 (58.31)	160 (47.76)	233 (68.73)		
Yes	281 (41.69)	175 (52.24)	106 (31.27)		
Smoking				*χ²* = 1.48	0.224
No	458 (67.95)	235 (70.15)	223 (65.78)		
Yes	216 (32.05)	100 (29.85)	116 (34.22)		
Alcohol				*χ²* = 0.55	0.460
No	519 (77.00)	262 (78.21)	257 (75.81)		
Yes	155 (23.00)	73 (21.79)	82 (24.19)		
Duration of Hypertension	0.00 (0.00, 6.00)	2.00 (0.00, 11.00)	0.00 (0.00, 1.00)	*Z* = -7.50	<0.001
Hb	142.00 (130.25, 152.00)	139.00 (129.00, 150.00)	143.00 (133.00, 153.00)	*Z* = -2.53	0.011
RDW	12.60 (12.20, 13.17)	12.70 (12.20, 13.20)	12.60 (12.15, 13.10)	*Z* = -1.74	0.083
HbA1C	8.30 (6.91, 10.00)	8.39 (7.01, 9.93)	8.10 (6.80, 10.15)	*Z* = -0.71	0.476
BUN	5.89 (4.90, 7.00)	6.04 (5.20, 7.37)	5.70 (4.70, 6.80)	*Z* = -3.82	<0.001
Scr	63.65 (54.10, 76.20)	65.30 (55.59, 80.06)	62.19 (52.00, 73.80)	*Z* = -3.22	0.001
SUA	303.95 (249.72, 366.12)	303.50 (253.05, 368.25)	304.90 (248.55, 362.10)	*Z* = -0.06	0.955
Alb	40.72 (38.51, 43.00)	40.00 (37.75, 42.55)	41.40 (39.50, 43.50)	*Z* = -4.62	<0.001
AST	20.74 (17.20, 27.90)	20.29 (17.30, 26.34)	20.90 (17.00, 29.21)	*Z* = -1.35	0.178
ALT	21.66 (15.82, 32.45)	20.30 (14.55, 28.85)	23.80 (17.20, 36.85)	*Z* = -3.72	<0.001
UACR(>30mg/g)	9.10 (5.42, 19.24)	11.00 (5.81, 57.41)	8.02 (5.25, 13.27)	*Z* = -5.14	<0.001
VitD	42.12 (30.68, 56.44)	43.02 (31.52, 58.91)	41.23 (29.70, 52.71)	*Z* = -2.45	0.014
OC	12.52 (9.66, 15.70)	11.82 (8.81, 15.05)	13.09 (10.59, 16.48)	*Z* = -4.09	<0.001
PTH	3.50 (2.69, 4.59)	3.29 (2.50, 4.32)	3.75 (2.85, 4.74)	*Z* = -3.57	<0.001
NLR	1.68 (1.27, 2.19)	1.86 (1.43, 2.56)	1.50 (1.14, 1.91)	*Z* = -8.25	<0.001
MLR	0.23 (0.19, 0.30)	0.26 (0.21, 0.34)	0.21 (0.18, 0.26)	*Z* = -7.89	<0.001
SIRI	0.85 (0.59, 1.21)	1.01 (0.67, 1.51)	0.72 (0.50, 0.99)	*Z* = -8.00	<0.001
SII	378.36 (282.59, 528.45)	413.68 (307.75, 628.79)	345.08 (256.66, 463.69)	*Z* = -5.81	<0.001
NE	3.62 (2.83, 4.41)	3.84 (3.04, 4.87)	3.41 (2.65, 4.20)	*Z* = -4.94	<0.001
LYM	2.09 (1.72, 2.59)	1.95 (1.58, 2.50)	2.24 (1.87, 2.73)	*Z* = -5.54	<0.001
MONO	0.50 (0.41, 0.61)	0.52 (0.42, 0.63)	0.48 (0.39, 0.58)	*Z* = -3.22	0.001
PLT	226.00 (196.00, 271.00)	222.00 (189.00, 262.50)	234.00 (205.00, 276.50)	*Z* = -2.62	0.009
AIP	0.24 (0.04, 0.46)	0.27 (0.08, 0.49)	0.20 (-0.01, 0.40)	*Z* = -3.70	<0.001
TyG	9.20 (8.70, 9.77)	9.30 (8.79, 9.98)	9.08 (8.56, 9.59)	*Z* = -4.44	<0.001
TyG-BMI	238.28 (209.50, 271.80)	246.68 (218.08, 281.60)	232.47 (203.82, 263.16)	*Z* = -4.51	<0.001
BMI	26.00 (23.00, 28.00)	26.00 (24.00, 29.00)	26.00 (23.00, 28.00)	*Z* = -2.91	0.004
FBG	7.33 (5.91, 9.33)	7.65 (6.00, 9.84)	7.08 (5.80, 8.84)	*Z* = -3.02	0.003
TG	1.63 (1.10, 2.50)	1.76 (1.19, 3.03)	1.54 (0.97, 2.26)	*Z* = -3.95	<0.001
TC	4.33 (3.51, 5.10)	4.36 (3.49, 5.22)	4.30 (3.55, 5.04)	*Z* = -0.52	0.604
HDL	0.97 (0.82, 1.13)	0.97 (0.81, 1.13)	0.97 (0.83, 1.12)	*Z* = -0.59	0.555
LDL	2.90 (2.27, 3.47)	2.95 (2.25, 3.49)	2.87 (2.29, 3.43)	*Z* = -0.63	0.528
DKD				*χ²* = 103.76	<0.001
No	585 (86.80)	246 (73.43)	339 (100.00)		
Yes	89 (13.20)	89 (26.57)	0 (0.00)		
DR				*χ²* = 573.96	<0.001
No	366 (54.30)	27 (8.06)	339 (100.00)		
Yes	308 (45.70)	308 (91.94)	0 (0.00)		
DPN				*χ²* = 511.98	<0.001
No	385 (57.12)	46 (13.73)	339 (100.00)		
Yes	289 (42.88)	289 (86.27)	0 (0.00)		

### 3.2 Based on LASSO regression for selecting predictive factors

**Figure 2:**
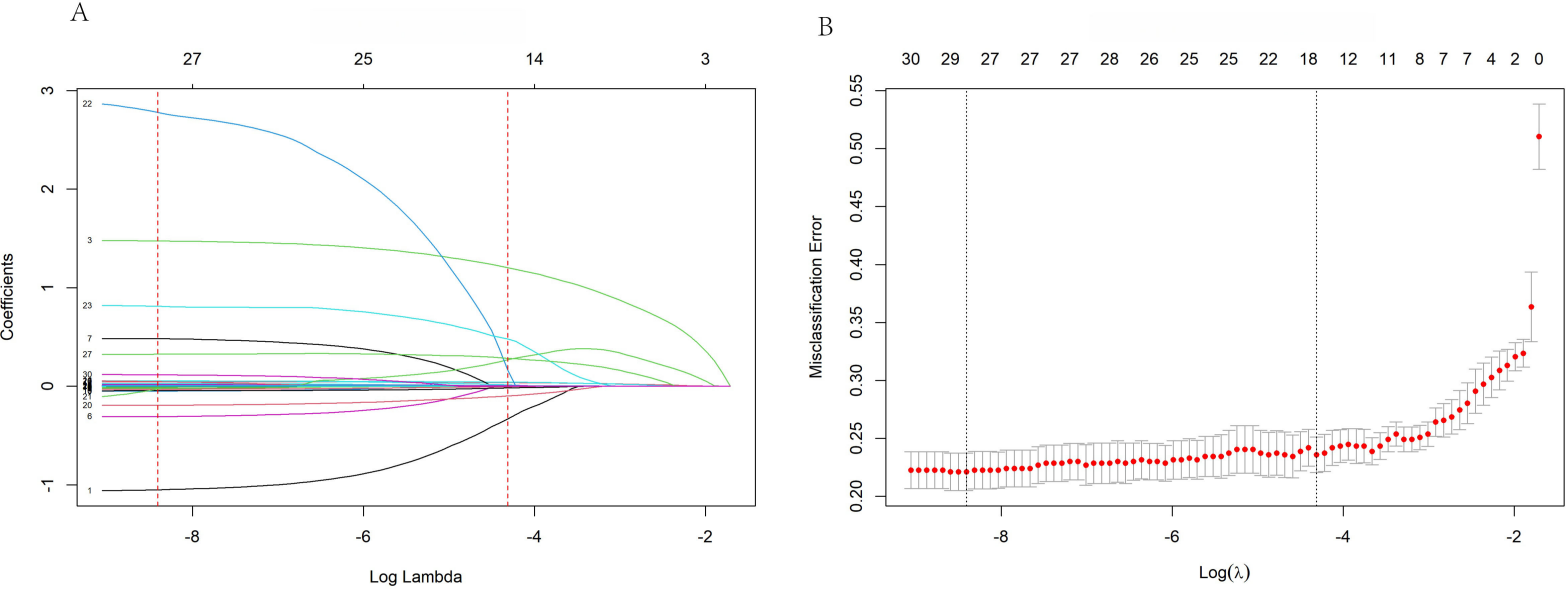
LASSO regression variable selection path plot (left) and cross-validation curve (right).

To filter predictive factors and prevent model overfitting, LASSO regression combined with 10-fold cross-validation was employed in the training set. When λ(1se)=0.01339904, a total of 17 core candidate variables were identified, including gender, age, duration of diabetes and hypertension, albumin, renal function indicators (urea, creatinine, uric acid, UACR), calcium-phosphorus metabolism indicators (vitamin D, osteocalcin, PTH), inflammatory indicators (NLR, MLR, SIRI), and metabolic indices (TyG, TyG-BMI). This filtering process simplified the model structure (**Figure 2**) while ensuring predictive efficacy, thereby enhancing clinical applicability.

### 3.3 Collinearity diagnosis

**Figure 3:**
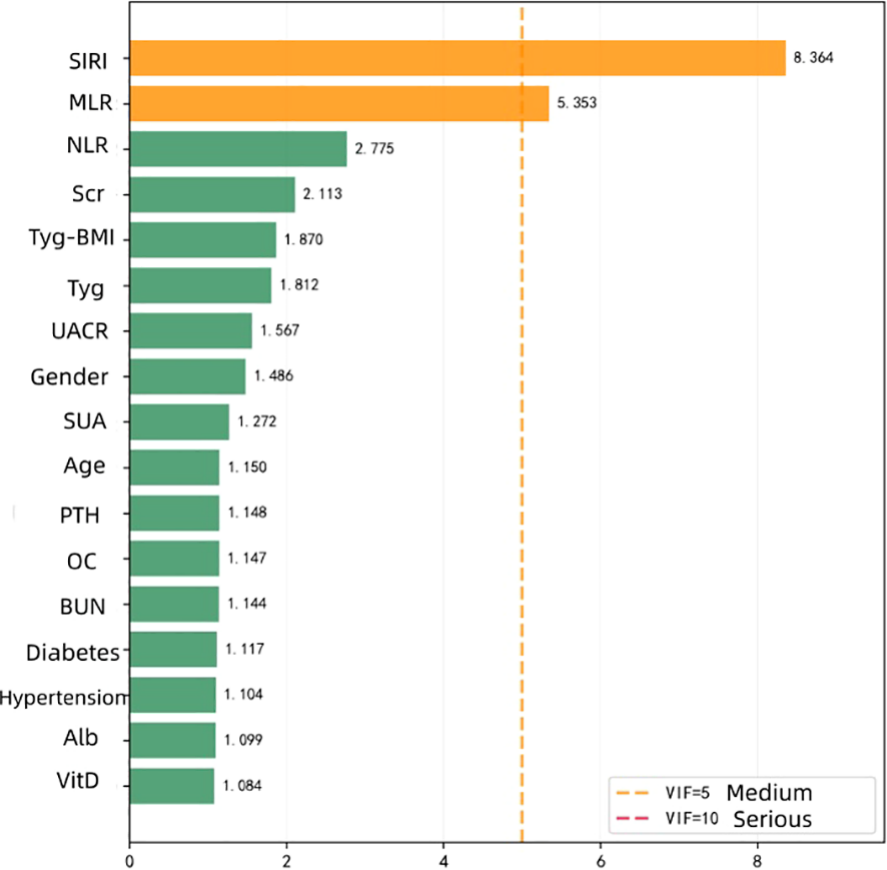
Bar chart of VIF analysis for multicollinearity of each variable.

**Figure 4:**
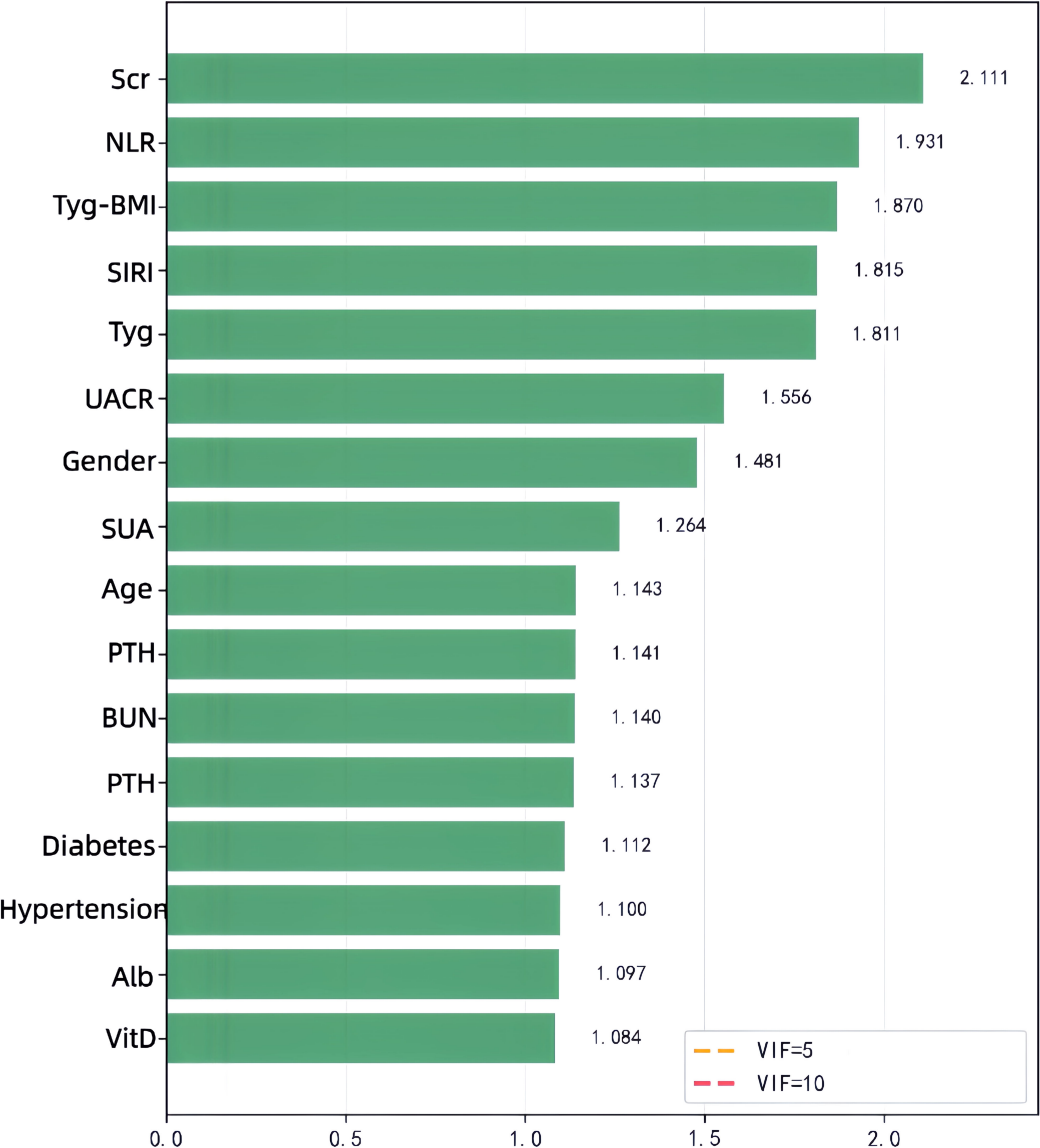
Bar chart showing the VIF analysis of multicollinearity for each variable after removing MLR.

To assess multicollinearity, this study calculated the variance inflation factor (VIF) for each variable. The initial analysis showed moderate collinearity between SIRI (8.364) and MLR (5.353) (**Table 4**; **Figure 3**). After removing the MLR related to the biological aspect of SIRI and recalculating, the VIFs of the remaining variables were all < 2.5 (e.g., TyG-BMI 1.870, TyG 1.81), indicating that the model did not have serious multicollinearity issues (**Table 5**; **Figure 4**). Ultimately, SIRI was retained while MLR was excluded from further analysis.

**Table 4:** Results of VIF analysis for candidate variables for multicollinearity.

Variable name	VIF value	Degree of collinearity
SIRI	8.364	Medium
MLR	5.353	Medium
NLR	2.775	No
Scr	2.113	No
TyG-BMI	1.87	No
TyG Index	1.812	No
UACR(>30mg/g)	1.567	No
Gender	1.486	No
SUA	1.272	No
Age	1.15	No
PTH	1.148	No
OC	1.147	No
BUN	1.144	No
Duration of diabetes	1.117	No
Duration of hypertension	1.104	No
Alb	1.099	No
VitD	1.084	No

**Table 5:** Results of VIF analysis for candidate variables for multicollinearity (after removing MLR).

Variable name	VIF value	Degree of collinearity
Scr	2.111	No
NLR	1.931	No
TyG-BMI	1.87	No
SIRI	1.815	No
TyG Index	1.811	No
UACR(>30mg/g)	1.556	No
Gender	1.481	No
SUA	1.264	No
Age	1.143	No
OC	1.141	No
BUN	1.14	No
PTH	1.137	No
Duration of diabetes	1.112	No
Duration of hypertension	1.1	No
Alb	1.097	No
VitD	1.084	No

### 3.4 Univariate and multivariate logistic regression analysis

This study uses the occurrence of microvascular complications of type 2 diabetes mellitus (T2DM) in the training cohort as a binary dependent variable (occurrence = 1, non-occurrence = 0). Candidate variables, selected through LASSO regression and collinearity processing, serve as independent variables. Univariate and multivariate logistic regression analyses are conducted sequentially to identify the independent risk factors for the occurrence of microvascular complications in T2DM, with results presented in **Table 6** and **Table 7**. Age, duration of diabetes, duration of hypertension, urea, creatinine, UACR (>30 mg/g), NLR, SIRI, TyG index, and TyG-BMI are positively correlated with the risk of complications, while albumin and osteocalcin are negatively correlated with the risk (**Table 6**). In the multivariate logistic regression analysis, variables with P<0.05 from the univariate analysis were included, utilizing a bidirectional regression method for selection. The final model revealed that age, duration of diabetes, duration of hypertension, UACR (>30 mg/g), SIRI, and TyG index are independent correlates of microvascular complications in type 2 diabetes mellitus (T2DM), while TyG-BMI did not reach statistical significance in the multivariate analysis (P = 0.070).

**Table 6:** Univariate regression analysis of influencing factors for microvascular complications in type 2 diabetes mellitus(T2DM)in the training set.

Variables	*B*	*S.E*	*Z*	*P*	OR (95%CI)
Gender					
Man					1.00
Woman	0.28	0.16	1.77	0.076	1.32 (0.97-1.80)
Age	0.05	0.01	7.15	<0.001	1.05 (1.04-1.07)
Duration of diabetes					
<10 years					1.00
≥10 years	1.61	0.18	9.09	<0.001	4.99 (3.53-7.06)
Duration of Hypertension	0.08	0.01	5.85	<0.001	1.08 (1.05-1.11)
BUN	0.14	0.04	3.27	0.001	1.15 (1.06-1.26)
Scr	0.02	0.00	4.48	<0.001	1.02 (1.01-1.03)
SUA	0.00	0.00	0.14	0.887	1.00 (1.00-1.00)
Alb	-0.05	0.02	-2.74	0.006	0.95 (0.92-0.99)
UACR(>30)	0.03	0.01	5.10	<0.001	1.03 (1.02-1.04)
VitD	0.01	0.00	1.92	0.054	1.01 (1.00-1.01)
OC	-0.03	0.01	-2.41	0.016	0.97 (0.94-0.99)
PTH	-0.05	0.04	-1.23	0.220	0.95 (0.88-1.03)
NLR	0.94	0.12	7.68	<0.001	2.57 (2.02-3.26)
SIRI	1.41	0.18	7.66	<0.001	4.10 (2.86-5.88)
TyG	0.50	0.10	5.21	<0.001	1.65 (1.37-1.99)
TyG-BMI	0.01	0.00	5.43	<0.001	1.01 (1.01-1.01)

OR, Odds Ratio; CI, Confidence Interval.

**Table 7:** Multivariate logistic regression analysis of influencing factors for microvascular complications in type 2 diabetes(T2DM)mellitus in the training set.

Variables	*β*	*S.E*	*Z*	*P*	OR (95%CI)
Intercept	-8.31	1.69	-4.90	<0.001	0.00 (0.00-0.01)
Age	0.06	0.01	5.94	<0.001	1.06 (1.04-1.08)
Duration of Diabetes					
<10 years					1.00
≥10 years	1.44	0.21	6.76	<0.001	4.20 (2.77-6.37)
Duration of Hypertension	0.05	0.01	3.31	<0.001	1.05 (1.02-1.08)
BUN	0.09	0.06	1.52	0.129	1.09 (0.98-1.22)
Alb	-0.04	0.02	-1.77	0.077	0.96 (0.92-1.00)
UACR(>30)	0.03	0.01	4.06	<0.001	1.03 (1.01-1.04)
OC	-0.03	0.02	-1.69	0.090	0.97 (0.93-1.00)
SIRI	1.06	0.22	4.87	<0.001	2.88 (1.88-4.40)
TyG	0.36	0.17	2.19	0.029	1.43 (1.04-1.98)
TyG-BMI	0.01	0.00	1.81	0.070	1.01 (1.00-1.01)

OR, Odds Ratio; C, Confidence Interval.

### 3.5 Evaluation of the efficacy of each indicator in predicting microvascular complications of type 2 diabetes mellitus

**Figure 5:**
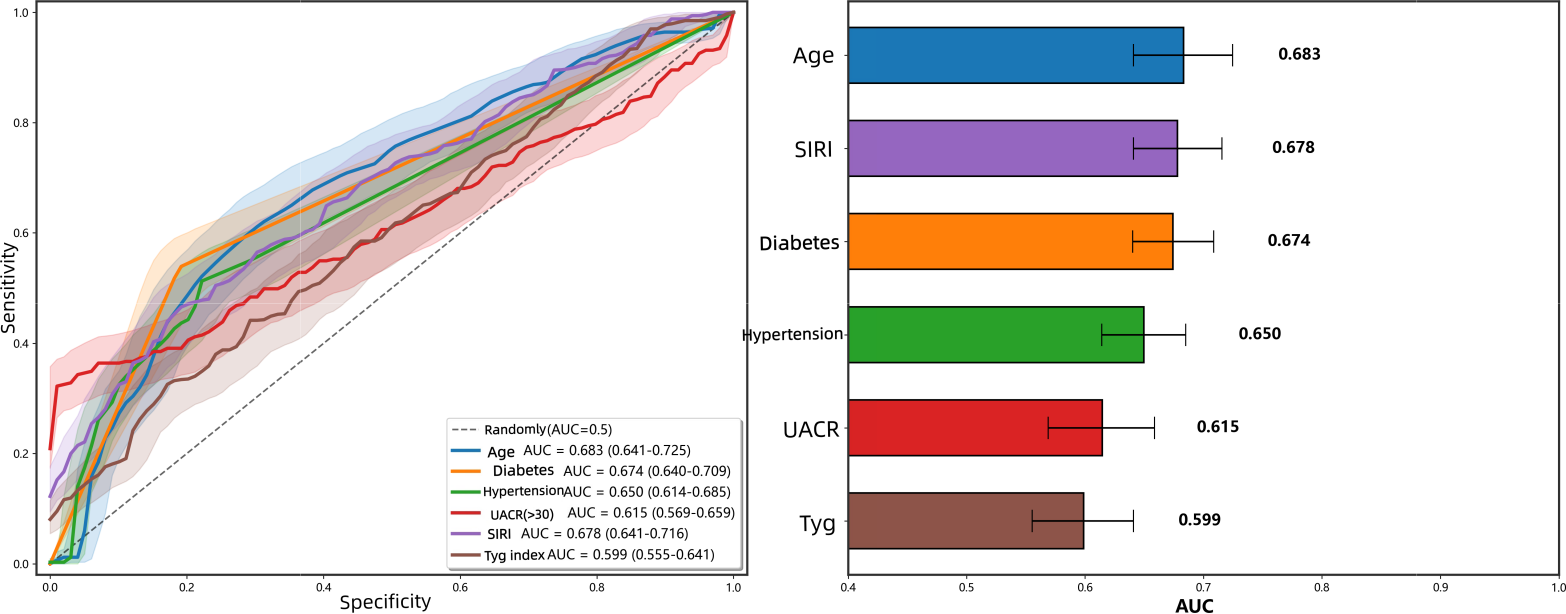
ROC curves of each indicator for predicting microvascular complications of type 2 diabetes mellitus.

To clarify the independent predictive efficacy of various risk factors for microvascular complications in Type 2 Diabetes Mellitus (T2DM), this study utilized training set data to plot the ROC curves for each indicator predicting T2DM microvascular complications, and calculated sensitivity, specificity, precision, F1 score, accuracy, and AUC values (95% CI), as shown in **Figure 5** and **Table 8**. The results indicate that the AUC for age is 0.683, with a corresponding sensitivity of 0.618, specificity of 0.690, and accuracy of 0.654 at the optimal cutoff value; the AUC for the duration of diabetes is 0.674, with sensitivity of 0.537, specificity of 0.811, and accuracy of 0.675; the AUC for the duration of hypertension is 0.650, with sensitivity of 0.510, specificity of 0.782, and accuracy of 0.647; the AUC for UACR (>30mg/g) is 0.615, with sensitivity of 0.322, specificity of 0.994, and accuracy of 0.660; the AUC for SIRI is 0.678, with sensitivity of 0.469, specificity of 0.805, and accuracy of 0.638; and the AUC for the TyG index is 0.599, with sensitivity of 0.325, specificity of 0.829, and accuracy of 0.579. Overall, while each individual indicator possesses a certain predictive capability, they all exhibit issues with low sensitivity or specificity, indicating moderate efficacy. This suggests that single indicators are insufficient for precise prediction of T2DM microvascular complications, necessitating the integration of core indicators to construct an inflammation-metabolism composite prediction model to enhance overall predictive efficacy.

**Table 8:** Efficacy indicators for predicting microvascular complications of type 2 diabetes mellitus.

Predictive indicators	Sensitivity	Specificity	Precision	F1 score	Accuracy rate	AUC	95%CI
Age	0.618	0.69	0.664	0.64	0.654	0.683	0.641-0.725
Duration of diabetes	0.537	0.811	0.738	0.622	0.675	0.674	0.640-0.709
Duration of hypertension	0.51	0.782	0.698	0.59	0.647	0.65	0.614-0.685
UACR(>30mg/g)	0.322	0.994	0.982	0.485	0.66	0.615	0.569-0.659
SIRI	0.469	0.805	0.704	0.563	0.638	0.678	0.641-0.716
TyG Index	0.325	0.829	0.653	0.434	0.579	0.599	0.555-0.641

### 3.6 Construction and validation of a prediction nomogram model for microvascular complications of type 2 diabetes mellitus

**Figure 6:**
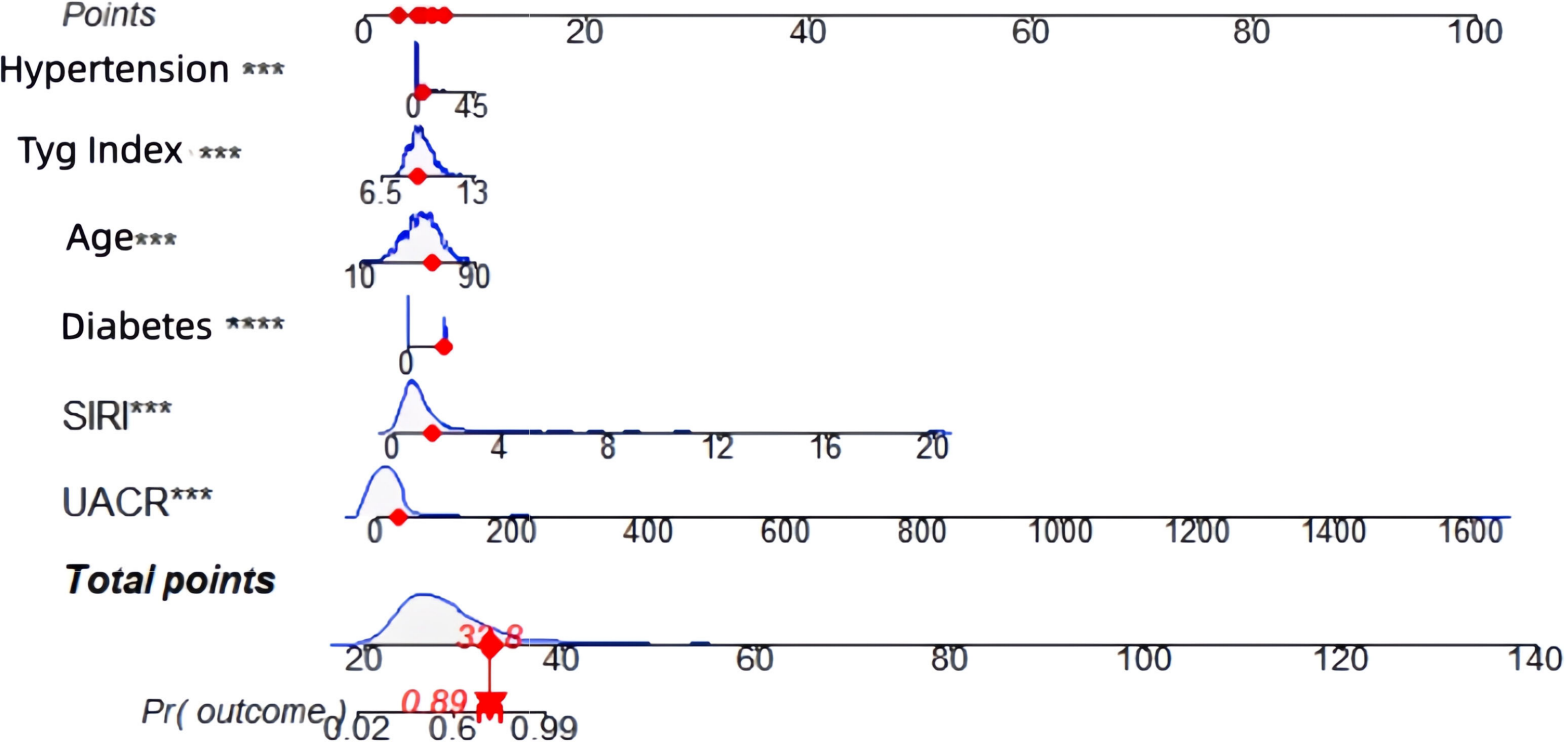
Prediction nomogram for microvascular complications of type 2 diabetes based on the combined inflammatory-metabolic indicators.

**Figure 7:**
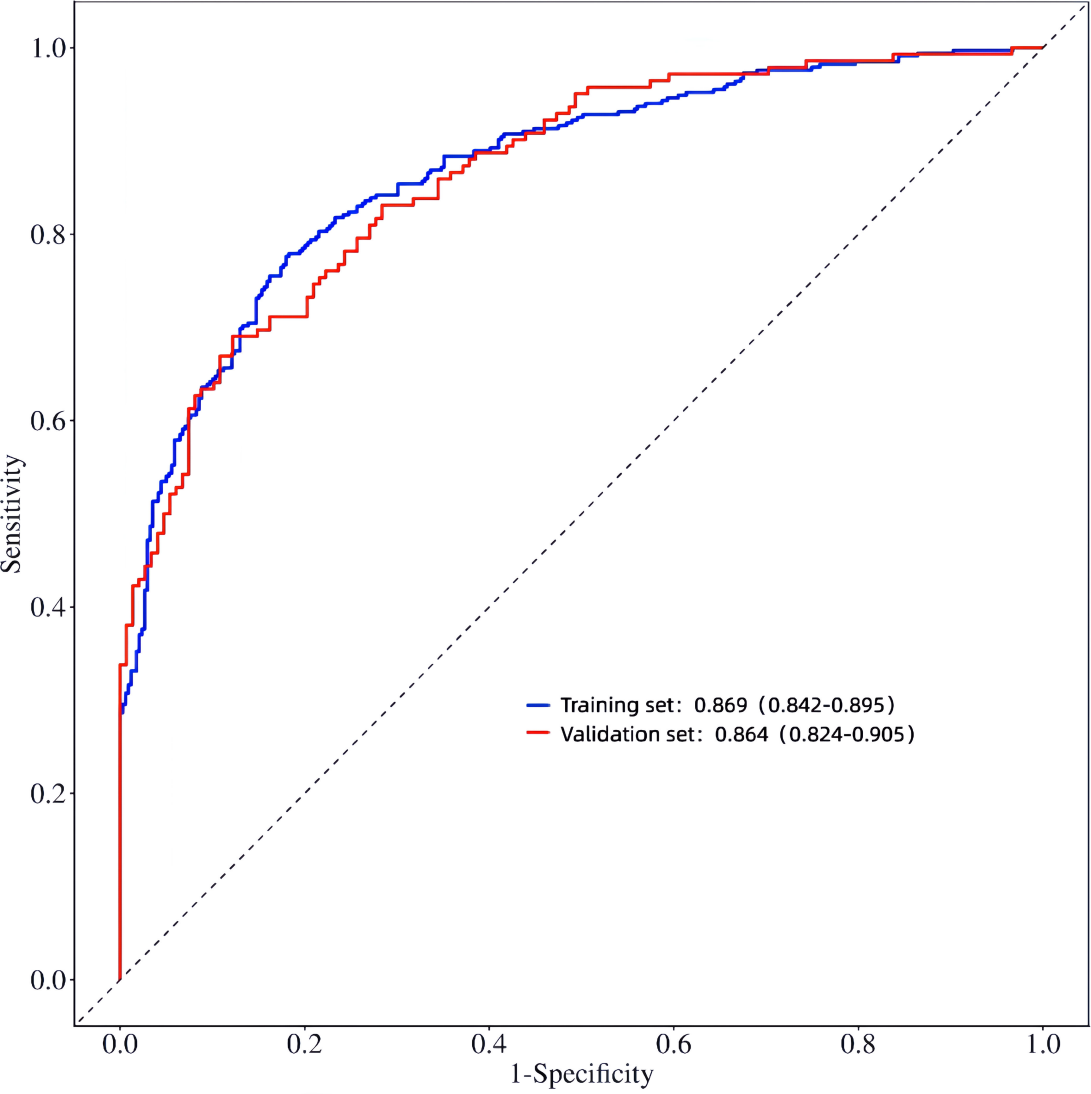
ROC curves of the line plot prediction model in the training set and validation set.

**Figure 8:**
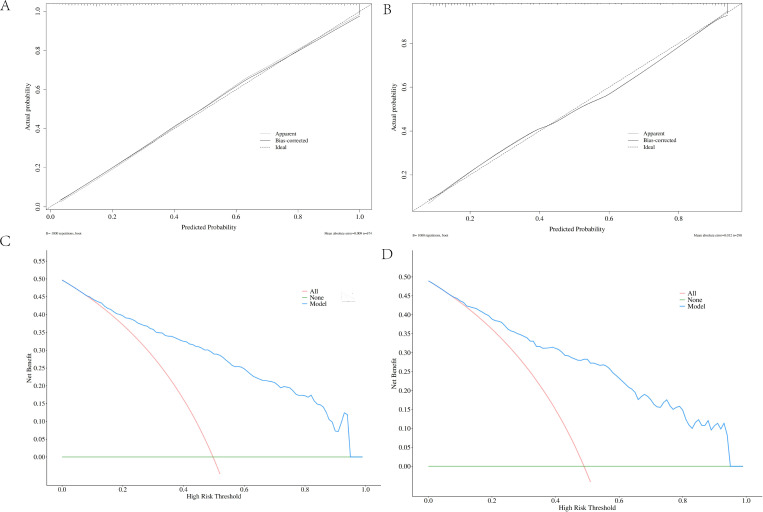
shows the calibration curve and DCA curve of the line graph prediction model. **(A, B)** Calibration curves of the model and validation groups; **(C, D)** DCA curves of the model and validation groups.

**Figure 9:**
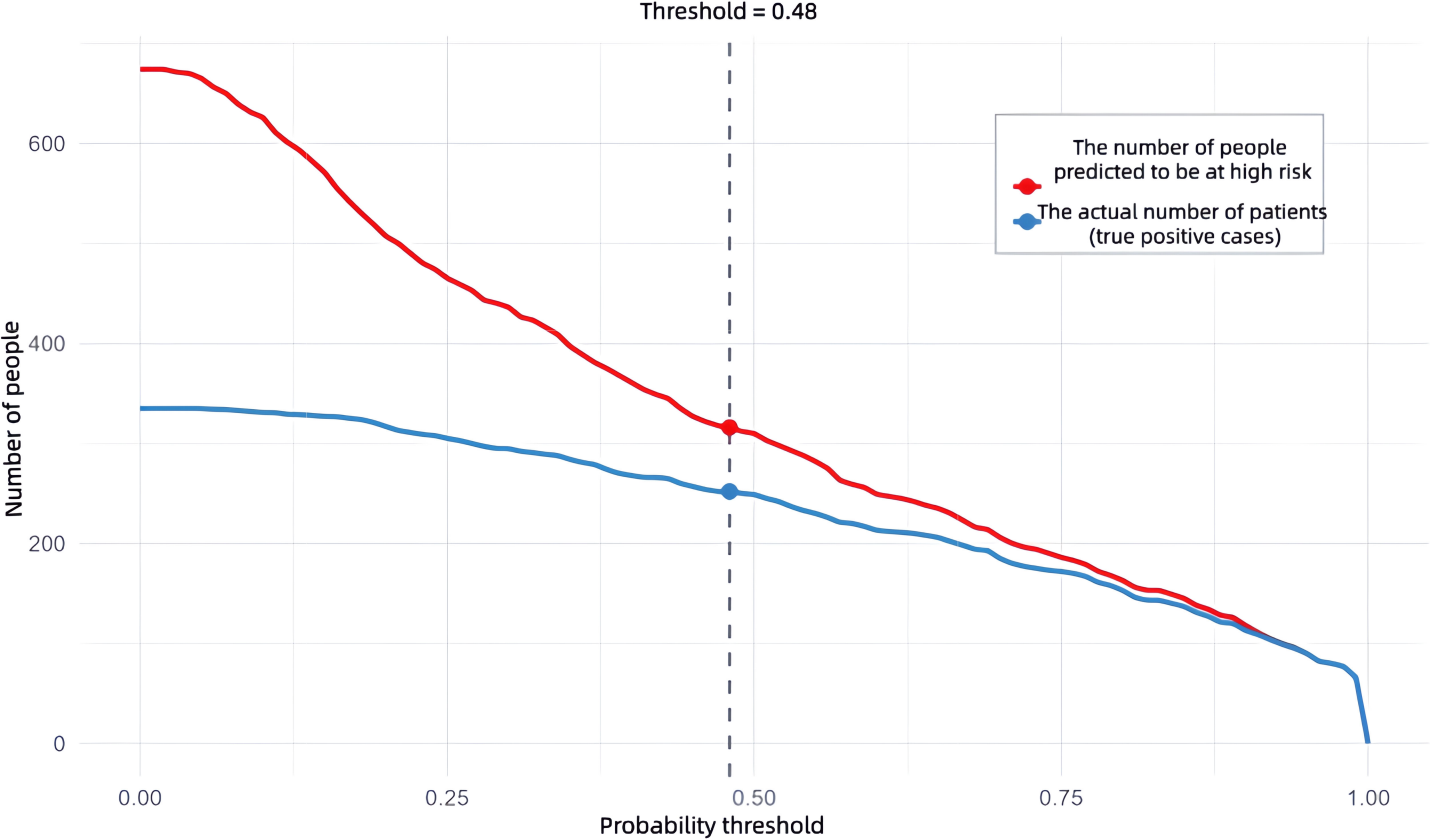
Shows the clinical impact curve of the line graph prediction model.

Based on the six independent risk factors (age, duration of diabetes, duration of hypertension, UACR (>30mg/g), SIRI, and TyG index) identified through multifactorial logistic regression analysis, this study constructed a nomogram model for predicting microvascular complications in type 2 diabetes mellitus (T2DM) based on inflammatory-metabolic composite indicators. The model was validated from three dimensions: predictive accuracy, calibration, and clinical net benefit using both training and validation sets. **Figure 6** presents the nomogram of this predictive model, where each indicator corresponds to a specific score based on actual clinical values. The total score is obtained by summing the scores of each indicator, which can then be used to directly read the probability of a patient developing microvascular complications of T2DM on the prediction probability axis. The figure presents a patient aged 60 years, with a diabetes duration of 1 year, hypertension duration of 5 years, a UACR of 30, a SIRI of 1.5, and a TyG index of 9.0. The patient’s risk of complications is 89.04%. To evaluate the overall predictive accuracy of the model, ROC curves for both the training and validation sets were plotted, as shown in **Figure 7**. The AUC value of the training set model was 0.869 (95% CI: 0.842-0.895), while the AUC value of the validation set model was 0.864 (95% CI: 0.824-0.905), both significantly higher than the AUC values of individual indicators. This suggests that the predictive efficacy of the composite inflammatory-metabolic index model is greatly enhanced, reaching an excellent level. Additionally, the AUC values of the training and validation sets were very close, with no significant statistical difference, indicating that the model has good stability. To validate the consistency between the model’s predicted probabilities and the actual occurrence probabilities, calibration curves were plotted, as shown in **Figure 8A** (calibration curve for the model group) and **Figure 8B** (calibration curve for the validation group). Both calibration curves closely align with the ideal diagonal line, showing no significant deviation, indicating that the model’s predicted probability of microvascular complications in T2DM is highly consistent with the clinically observed occurrence probabilities, demonstrating excellent calibration of the model (Hosmer-Lemeshow test P values were 0.180 and 0.614, respectively). To assess the clinical utility of the model, decision curve analysis (DCA) curves were plotted, as illustrated in **Figure 8C** (DCA curve for the model group) and **Figure 8D** (DCA curve for the validation group). The net benefit curves of the model are significantly higher than both the “all curve” and the “no intervention curve,” and maintain a positive net benefit across a wide range of risk thresholds. This suggests that the nomogram model can effectively identify high-risk patients for microvascular complications in T2DM in clinical practice, providing a reliable quantitative basis for early clinical intervention and the development of personalized treatment plans, thus possessing substantial clinical utility and significance for promotion. In this study, a good balance between sensitivity and specificity was achieved, ranging from 0.48, as shown in **Figure 9**.

## 4 Discussion

### 4.1 The pathological mechanism and clinical risks of diabetic microvascular complications

Diabetic microangiopathy (DMC) has long been regarded as a metabolic disorder resulting from sustained hyperglycemia. Hyperglycemia can induce oxidative stress and endothelial dysfunction through the polyol pathway, PKC activation, and AGE-RAGE signaling, leading to basement membrane thickening and altered capillary permeability (20). This ultimately results in retinal damage, disruption of the glomerular filtration barrier, and insufficient microcirculation perfusion in the nerves (21–23).

Increasing evidence suggests that low-grade chronic inflammation is an important synergistic factor driving microvascular damage (24). Inflammation can enhance leukocyte adhesion, promote microthrombosis, and interact with lipid metabolism disorders and insulin resistance to accelerate the structural damage of microvessels. Furthermore, insulin resistance exacerbates the burden on vascular walls and glomeruli through hyperinsulinemia and lipotoxicity (25, 26). Therefore, integrating the ratio of peripheral blood inflammatory cells with metabolic indicators through a multi-dimensional mechanism of inflammation-immunity-metabolism has clear biological rationale (27).

Microvascular lesions are characterized by insidious onset, early asymptomatic phases, and often irreversible damage; once symptoms such as visual impairment, proteinuria, or sensory abnormalities appear, the condition has usually progressed to the moderate or late stage (27). Long-term hyperglycemia, prolonged diabetes duration, hypertension, lipid metabolism disorders, and insulin resistance are all recognized risk factors (28). However, their predictive ability when considered individually is limited, indicating the need for a risk assessment tool that integrates multi-dimensional indicators (29). Current clinical risk identification still faces shortcomings: traditional indicators such as HbA1c or blood lipids only reflect a single metabolic dimension, making it difficult to capture the synergistic effects of inflammation and metabolism (30). Additionally, the insufficient ability to identify high-risk individuals early limits precise interventions. Therefore, developing predictive tools that integrate inflammatory and metabolic composite indicators holds significant clinical value.

### 4.2 The research results are compared with those of previous studies

A substantial body of research has confirmed that hyperglycemia, insulin resistance, hypertension, and lipid metabolism disorders are closely associated with Diabetic Microvascular Complications (DMC) (31, 32). However, traditional single indicators, particularly HbA1c, have limited predictive efficacy (33). This study integrates inflammatory and metabolic composite indicators based on LASSO-Logistic regression, further validating the advantages of composite indicators in risk assessment.

#### 4.2.1 Hypertension and DMC

Hypertension is an important factor promoting the progression of Diabetic Retinopathy (DR) and Diabetic Kidney Disease (DKD) (34). The mechanisms involved include endothelial dysfunction, enhanced oxidative stress, and amplified inflammatory responses (35). The results of this study are consistent with previous findings, showing a significant correlation between hypertension and DMC risk, which may be exacerbated by the interplay between inflammation and metabolism, thereby aggravating microvascular injury (36, 37). Notably, the interaction between SIRI and TyG-BMI suggests that abnormal blood pressure may enhance risk effects through multiple pathways.

#### 4.2.2 SIRI and DMC

SIRI comprehensively reflects changes in neutrophils, monocytes, and lymphocytes (38), demonstrating advantages in assessing overall inflammation levels (39). Previous studies have shown that inflammation contributes to the occurrence of DMC through endothelial dysfunction, oxidative stress, and immune system activation (40). Previous studies have indicated that inflammatory factors such as CRP and TNF-α are associated with both DR and DKD (41, 42). This study finds that SIRI can effectively predict DMC, outperforming traditional single inflammatory indicators, further supporting the critical role of inflammation in microvascular lesions.

#### 4.2.3 tyg and DMC

The TyG index, as an ideal alternative indicator for insulin resistance (IR), demonstrates superior potential compared to the traditional HOMA-IR in assessing microvascular complications in type 2 diabetes mellitus (T2DM) (43). Insulin resistance is the core pathological basis of T2DM and its microvascular complications, while a high TyG index reflects a sustained stress state induced by prolonged exposure to dual metabolic toxicity of ‘high glucose-high fat’. Compared to single blood glucose or lipid indicators, the TyG index can more comprehensively characterize the cumulative damage to the microvascular wall caused by dysregulation of glucose and lipid metabolism (44).

#### 4.2.4 Advantages of composite indicators and multi-factor synergy

This study found that although individual indicators such as age, SIRI, disease duration, and TyG index are significantly correlated with complications, their independent predictive accuracy is limited. The risk increases sharply for those with a disease duration exceeding 10 years, reflecting the ‘metabolic memory’ effect: the oxidative stress and endothelial damage induced by prolonged hyperglycemia have a cumulative nature, and even if blood glucose levels are normalized later, the pathological process may continue. Since non-intervenable factors such as age and disease duration cannot reflect the dynamic evolution of the internal environment in real time, integrating dynamic indicators becomes particularly important. Microvascular complications are the result of the interplay between metabolic disorders and chronic inflammation (45). The inflammation-metabolism composite model constructed through LASSO regression systematically integrates pathological information from different dimensions, such as SIRI (immune activation) and TyG index (insulin resistance). Validation results show that this model significantly enhances sensitivity while maintaining high specificity, effectively reducing the missed diagnosis rate of high-risk individuals and providing a more practically valuable quantitative tool for early clinical identification and precise intervention.

This study confirms the importance of inflammatory and metabolic composite indicators in predicting the risk of DMC, which is highly consistent with previous evidence. Compared to traditional single indicators, composite indicators such as SIRI and TyG can more comprehensively reflect the patients’ inflammatory and metabolic load, thereby improving the accuracy of risk prediction. The predictive model constructed by integrating multidimensional biological mechanisms provides a preliminary and potential framework for the early identification and personalized intervention of microvascular complications in type 2 diabetes mellitus (T2DM).

### 4.3 Research advantages and limitations

#### 4.3.1 Research advantages

(i)Integrating inflammation and metabolism into a dual-dimensional predictive system that aligns more closely with pathological mechanisms. This study incorporates multiple composite indicators of inflammation (NLR, MLR, SII, SIRI) and metabolism (AIP, TyG, TyG-BMI), covering key pathological aspects such as inflammatory response, immune imbalance, and metabolic disorders. Compared to previous studies that focused solely on single indicators, this approach better reflects the multidimensional mechanisms of DMC. All indicators are derived from routine blood tests, lipid profiles, and blood glucose checks, ensuring high clinical accessibility and promotional value. (ii) Employing rigorous variable selection and modeling strategies. LASSO regression has advantages in handling multiple variables and potential multicollinearity, effectively selecting key features to construct a more robust and concise model. Subsequently, multivariable logistic regression is used for modeling, enhancing the applicability and stability of the predictive tool. Such methods have been widely recognized in chronic disease risk prediction research. (iii) Adequately adjusting for traditional risk factors to improve model credibility. This study includes common risk factors such as age, gender, disease duration, renal function, and BMI in the model construction to reduce confounding effects. Combined with methods like cross-validation, this can further enhance the robustness and generalizability of the model. (iv) Covering a larger sample and multiple types of microvascular complications. This study simultaneously evaluates three types of microvascular complications: diabetic retinopathy (DR), diabetic kidney disease (DKD), and diabetic peripheral neuropathy (DPN). This approach overcomes the limitations of previous studies that focused solely on single complications, facilitating a more comprehensive exploration of the general predictive value of indicators and potential organ-specific differences.

#### 4.3.2 Research limitations

(i) Limitations of Causal Inference in Retrospective Design: This study employs a retrospective design, making it challenging to accurately determine causal relationships. It can only indicate the association between the indicators and DMC, without concluding that a high SIRI or high TyG causes the occurrence of microvascular complications. While predictive models are valuable, they do not equate to the validation of causal chains. Further confirmation is required through prospective cohorts, time-dependent analyses (such as Cox regression), or interventional studies. (ii) The single-center study affects external generalizability. The research data are sourced from a single medical institution, and the sample may exhibit concentration in age structure, treatment strategies, and lifestyle factors, limiting the extrapolation of results to different regions and healthcare systems. Future research should be conducted in a multi-center setting for validation. Most importantly, the model remains a preliminary framework and cannot be recommended for widespread clinical application until its reliability and generalizability are rigorously verified in large-scale, multi-center external populations. (iii) Biological variability and confounding factors may affect indicator stability. Peripheral blood cell counts are easily influenced by infections, inflammation, stress, and medications, while lipid and blood glucose levels are also affected by diet, medication, and liver and kidney function. If sampling and exclusion criteria are not consistently applied, it may lead to fluctuations in indicators and reduce model stability. (iv)The lack of dynamic data makes it difficult to reflect disease progression characteristics. This study is based on single test results, whereas DMC is a chronic progressive disease, and its inflammatory and metabolic levels fluctuate with treatment and disease course changes. The absence of longitudinal monitoring data may underestimate or overestimate the true predictive ability of certain indicators.(v)although we implemented strict exclusion criteria to eliminate the influence of potent anti-inflammatory and metabolism-altering medications (e.g., glucocorticoids and immunosuppressants), the possibility of residual confounding remains. Individual variations in response to baseline glucose-lowering or lipid-lowering therapies, as well as the use of over-the-counter supplements not fully captured in electronic records, might still exert subtle effects on the measured inflammatory and metabolic markers.

## Data Availability

The raw data supporting the conclusions of this article will be made available by the authors, without undue reservation.
